# Survival analysis of heart failure patients: A case study

**DOI:** 10.1371/journal.pone.0181001

**Published:** 2017-07-20

**Authors:** Tanvir Ahmad, Assia Munir, Sajjad Haider Bhatti, Muhammad Aftab, Muhammad Ali Raza

**Affiliations:** Department of Statistics, Government College University, Faisalabad, Pakistan; Azienda Ospedaliero Universitaria Careggi, ITALY

## Abstract

This study was focused on survival analysis of heart failure patients who were admitted to Institute of Cardiology and Allied hospital Faisalabad-Pakistan during April-December (2015). All the patients were aged 40 years or above, having left ventricular systolic dysfunction, belonging to NYHA class III and IV. Cox regression was used to model mortality considering age, ejection fraction, serum creatinine, serum sodium, anemia, platelets, creatinine phosphokinase, blood pressure, gender, diabetes and smoking status as potentially contributing for mortality. Kaplan Meier plot was used to study the general pattern of survival which showed high intensity of mortality in the initial days and then a gradual increase up to the end of study. Martingale residuals were used to assess functional form of variables. Results were validated computing calibration slope and discrimination ability of model via bootstrapping. For graphical prediction of survival probability, a nomogram was constructed. Age, renal dysfunction, blood pressure, ejection fraction and anemia were found as significant risk factors for mortality among heart failure patients.

## Introduction

Heart failure is the state in which muscles in the heart wall get fade and enlarge, limiting heart pumping of blood. The ventricles of heart can get inflexible and do not fill properly between beats. With the passage of time heart fails in fulfilling the proper demand of blood in body and as a consequence person starts feeling difficulty in breathing.

The main reason behind heart failure include coronary heart disease, diabetes, high blood pressure and other diseases like HIV, alcohol abuse or cocaine, thyroid disorders, excess of vitamin E in body, radiation or chemotherapy, etc. As stated by WHO [1] Cardiovascular Heart Disease (CHD) is now top reason causing 31% of deaths globally. Pakistan is also included in the list of countries where prevalence of CHD is increasing significantly. According to report by Al-Shifa hospital [2], 33% of Pakistani population above 45 has hypertension, 25% of patients over 45 years suffer diabetes mellitus, and CHD deaths in Pakistan has reached about 200,000 per year i.e. 410/100,000 of the population). All this results in increased prevalence of heart failure. Rate of heart failure patients in Pakistan is estimated to be 110 per million [1]. Rising stress of economic and social issues in the modern era, greasy food with little exercise results towards increased prevalence of heart failure in Pakistan.

Despite of this alarming situation, Pillai and Ganapathi [3] have reported that there are no reliable estimates of heart failure incidence and prevalence in this region while they are required due to poor and oily diet, lack of exercise and poor health care policies in this region. There are some projections based on prevalence data only from western countries.

In addition to relative scarcity of studies focusing on heart failure in this region, the present study has specific importance in the Pakistani context, as diet patterns in Pakistan are different with other the countries of South Asia like India, Bangladesh, Nepal and Sri Lanka.

The main objective of this study is to estimate death rates due to heart failure and to investigate its link with some major risk factors by choosing Faisalabad (third most populous city of Pakistan) as study area.

## Methods

### Detail of data

Current study is based on 299 patients of heart failure comprising of 105 women and 194 men. All the patients were more than 40 years old, having left ventricular systolic dysfunction and falling in NYHA class III and IV. Follow up time was 4–285 days with an average of 130 days. Disease was diagnosed by cardiac echo report or notes written by physician. Age, serum sodium, serum creatinine, gender, smoking, Blood Pressure (BP), Ejection Fraction (EF), anemia, platelets, Creatinine Phosphokinase (CPK) and diabetes were considered as potential variables explaining mortality caused by CHD. Age, serum sodium and CPK are continuous variables whereas EF, serum creatinine and platelets were taken as categorical variables. EF was divided into three levels (i.e. EF≤30, 30<EF≤45 and EF>45) and platelets was also divided into three level on the basis of quartiles. Serum creatinine greater than its normal level (1.5) is an indicator of renal dysfunction. Its effect on mortality was studied as creatinine >1.5 vs ≤1.5. Anemia in patients was assessed by their haematocrit level. Following McClellan et al. [4] the patients with haematocrit less than 36 (minimum normal level of haematocrit) were taken as anemic. The information related to risk factors were taken from blood reports while smoking status and blood pressure were taken from physician’s notes.

The study was approved by Institutional Review Board of Government College University, Faisalabad-Pakistan and the principles of Helsinki Declaration were followed. Informed consent was taken by the patients from whom the information on required characteristics were collected/accessed.

### Statistical techniques

Due to the presence of censored data, survival analysis was used to estimate the survival and mortality rates. Kaplan & Meier [5] product limit estimator was used to make comparisons between survival rates at different levels explanatory variables. Cox regression as presented by Collett [6] was used to develop a model that can link the hazard of death for an individual with one or more explanatory variables and test the significance of these variables.

Let hazard of death depends on *p* explanatory variables *X*_1_, *X*_2_, ⋯ *X*_*p*_ then the hazard function for *i*^*th*^ individual can be defined by Cox model as

$$h_{i}(t)=h_{0}(t)\;e^{\beta_{1}X_{1i}+}{{}^{\beta_{1}X_{1i}+\ldots +}}^{\beta_{1}X_{1i}}$$

For determining the functional form of any particular independent variable following Fitrianto & Jiin [7] and Gillespie [8], plot of Martingale residuals versus different values (or levels) of a variable were used. The functional form of CPK was not linear therefore it was log transformed.

Following Pavlou et al. [9] model validation was assessed by bootstrapping [10–12] with 200 bootstrap replications. Internal validation of model was further checked by calculating calibration slope [13] for the average linear predictor. The calibration slope helped in estimating the ability of model for survival probability prediction. Discriminating ability of model was assessed by ROC curve [14]. A nomogram [15] was also built to predict the survival probabilities graphically.

## Results

Up to end of follow-up period, 96 (32%) patients died due to CHD. Table 1, presents different baseline characteristics of dead and censored patients at the end of follow up period.

**Table 1:** Baseline characteristics for dead and censored patients.

Continuous Variables			Categorical Variables		
Variable	Dead (96)	Censored (203)	Variable	Dead (96)	Censored (203)
Creatinine	1.83	1.18	Male	62 (64%)	132 (65%)
Sodium	135.39	137.22	Smoking	30 (31%)	66 (32%)
CPK	670	540	Diabetes	40 (42%)	85 (42%)
Age	65.21	58.76	BP	40 (42%)	66 (32%)
Platelets	256381	266657	Anemia	54 (56%)	83 (40%)
EF	33.46	40.267			

The results of Cox regression model are presented in Table 2. As Cox regression is semi parametric model, hence estimate of intercept (baseline hazard) was not provided by model fitting. According to Cox model, age was most significant variable.

**Table 2:** Significance of variables under Cox regression.

Variable	β-coefficient	HR	Z-value	P-value
Age	0.0462	1.0473	4.81	0.0000
Gender	-0.1978	0.8205	-0.80	0.4239
EF≤30 vs 30<EF≤45	-1.1068	0.3306	-4.36	0.0000
EF≤30 vs EF>45	-0.8894	0.4109	-3.18	0.0015
Smoking	0.1928	1.2127	0.77	0.4431
Diabetes	0.0992	1.1043	0.45	0.6553
Blood pressure	0.5043	1.6558	2.33	0.0195
Serum creatinine	0.8051	2.2368	3.01	0.0026
Serum sodium	-0.0658	0.9363	-2.79	0.0052
log(CPK)	0.1444	1.1554	1.41	0.1596
Anemia	0.5709	1.7699	2.59	0.0096
Platelets(≤Q_1_)	0.4054	1.4999	1.62	0.1042
Platelets(≥Q_3_)	0.3926	1.4800	1.46	0.1446

**Fig 1:**
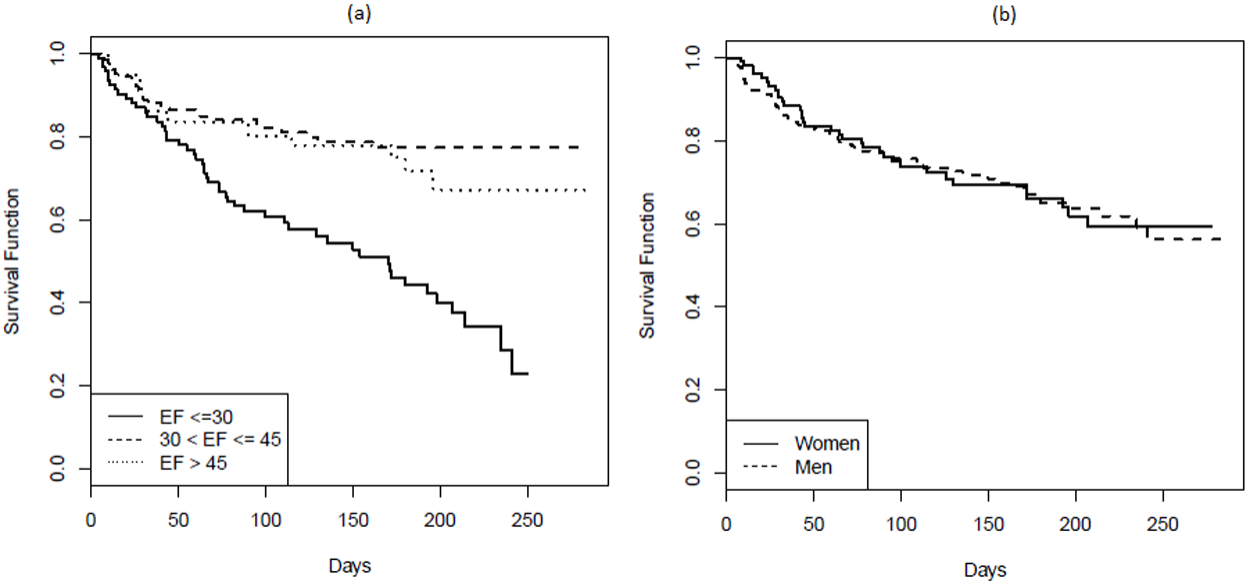
(a) Kaplan Meier curves were used to study the survival at different levels of EF and in part (b), survival curves of male and female.

Coefficient concerning age indicated that chances of death due to CHD increase with growing age. Hazard of death due to CHD increases by 4% for every additional year of age. EF was another significant factor, hazard rate among patients with EF ≤30 was 67% and 59% higher as compared to the patients with 30<EF≤45 and EF≥45 respectively. In Fig 1(a), Kaplan Meier survival curve was constructed for each level of EF. It is obvious that survival for EF ≤30 was lower than other two levels. Moreover, relatively small difference between the survival of patients with 30<EF<45 and EF≥45 can be observed. Serum creatinine was significant with p-value = 0.0026. It means death hazard gets more than double for unit increase in Serum creatinine. Serum sodium was significant with p-value = 0.0052 and its one unit (meq/L) increase decreases the hazard by 6%. Anemia was significant variable with p-value = 0.0096 and an anemic patient had 76% more chances of death as compared to non-anemic patient. According to results in Table 2, gender, smoking, diabetes, CPK and platelets were found to be non-significant.

Ejection fraction is an important measurement of how well one’s heart is pumping and is used to help classify heart failure and guide treatment. The EF is also found to be significant correlate of deaths among heart failure patients from Cox regression for present sample. Keeping its importance in view, EF is further analyzed through baseline characteristics (Table 3) and Kaplan Meier curves (Fig 1(a)) which shows similar pattern as presented in Cox regression results.

**Table 3:** Baseline characteristics with respect to EF levels.

	Variable	Dead (96)			Censored (203)		
		EF≤30 (51)	30<EF<45 (26)	EF≥45 (19)	EF≤30 (42)	30<EF<45 (100)	EF≥45 (61)
**Continuous Variables**	Creatinine	1.5447	2.225	2.084	1.345	1.122	1.179
	Sodium	134.76	135.73	136.53	136.6	137.23	137.59
	CPK	601	923	511	568	614.8	398.6
	Age	61.84	68.92	69.21	56.95	58.59	60.30
	Platelets	258577	260863	244353	256937	261280	282166
**Categorical Variables**	Male	35 (69%)	18 (69%)	09 (47%)	30 (71%)	63 (63%)	39 (64%)
	Smoking	17 (33%)	09 (35%)	04 (21%)	15 (36%)	32(32%)	19(31%)
	Diabetes	20 (39%)	20 (38%)	10 (53%)	18 (43%)	47(47%)	20(33%)
	BP	20 (39%)	12 (46%)	07 (37%)	14 (33%)	26 (26%)	26 (43%)
	Anemia	25 (49%)	11 (42%)	10 (53%)	18 (43%)	38 (38%)	27 (44%)

In Fig 1(b), Kaplan Meier survival curves were constructed for both genders showed almost identical survival pattern.

### Model validation

For model validation, calibration slope and ROC curve are developed from 200 bootstrapped samples. Calibration slope was equal to 0.96, which showed that model was not over fitted and predictions made by this model would neither be overestimated nor under estimated.

**Fig 2:**
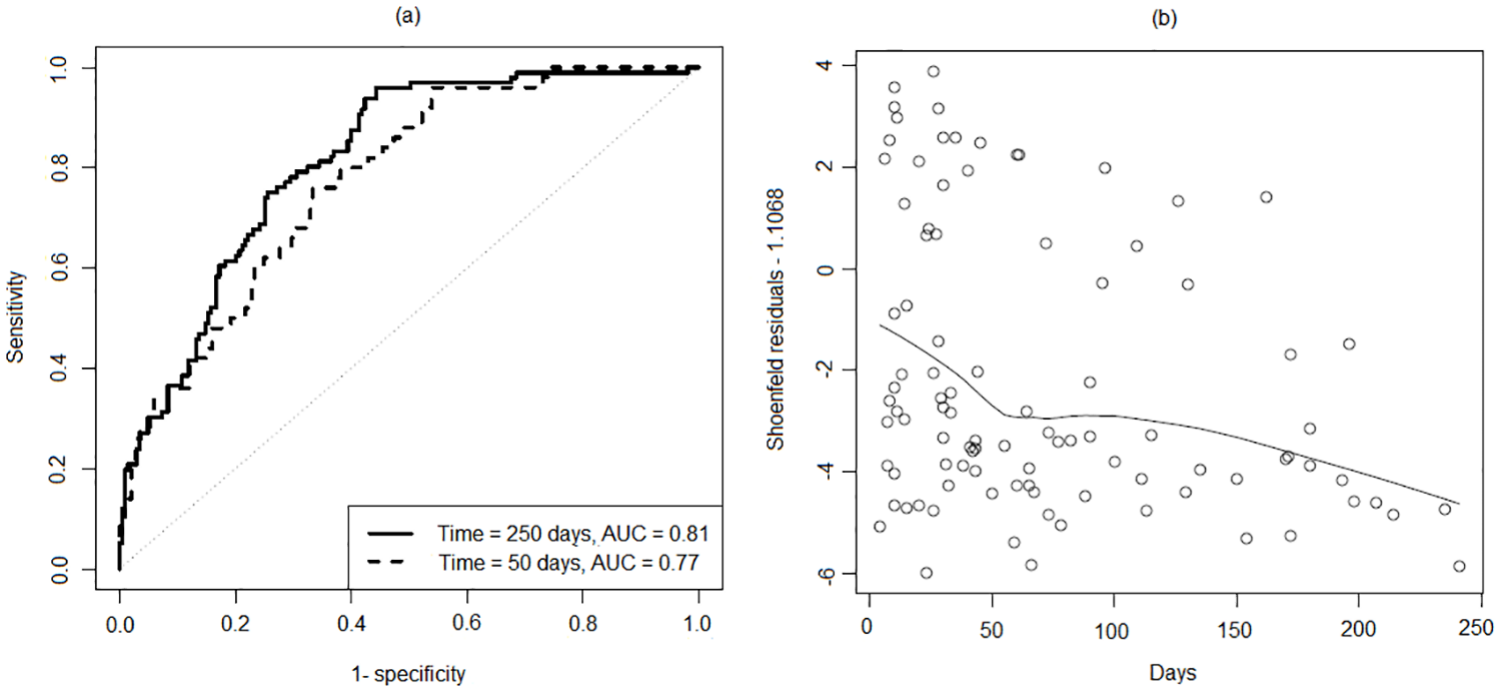
(a) ROC curves for discriminant ability w.r.t time (b) Effect of EF w.r.t time.

Discrimination ability was checked by ROC curve in Fig 2(a). Area under the curve (AUC) was 0.81 at time of 250 days and 0.77 at time of 50 days thus it can be interpreted that the model was able to correctly recognize the event of death for 81% and 77% patients within 250 and 50 days respectively. It shows that discrimination ability of Cox model is higher at longer follow up time. The reason of this difference may be due to the violation of constant effect assumption of EF which is evident in Fig 2(b) which displays that effect of EF increases with the passage of time. As EF is highly significant for mortality (see Table 2), hence with passage of time model’s discrimination ability increases.

### Nomogram for prediction

**Fig 3:**
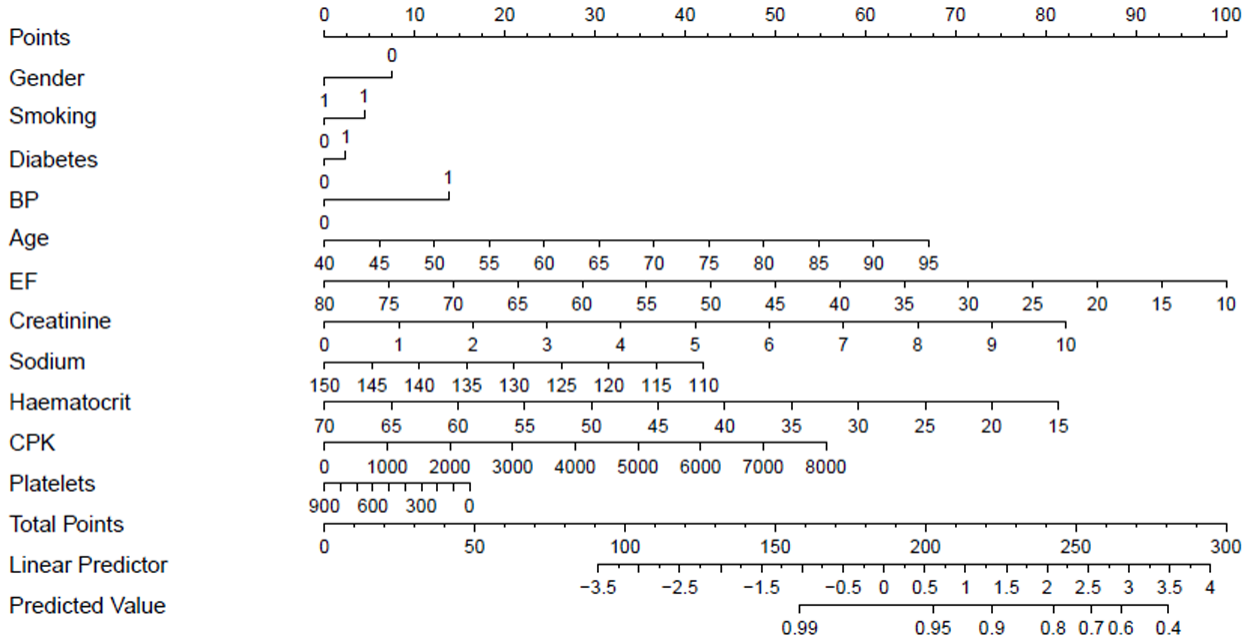
Nomogram for survival probability.

Calibration slope and discrimination ability suggested that Cox model is able to predict probability of survival and hazard sufficiently. On the basis of these results, nomogram is presented in Fig 3 to provide the graphical predictions of probability after assigning different points to each independent variable with respect to their significance. Sum of these points provides an estimate of probability of survival.

For example, an 80 year old non-smoker female diabetic patient with high blood pressure, EF = 40, haematocrit = 35, sodium = 140, creatinine = 5.2, platelets = 300 thousands and CPK = 3000 have points equal to 50+0+8+3+14+56+50+12+40+10+20 = 263 and probability of her survival is 0.60. The Cox model used for constructing this nomogram was fitted on original values of variables.

## Discussion

The statistical analysis identified age, EF, creatinine, sodium, anemia and BP as the significant variables affecting the likelihood of mortality among heart failure patients. Most of studies [16–17] supported the male gender as predictor of mortality among heart failure patients. However, like Román et al. [18] in this study odd ratio of men/women is not significant. With respect to significance and importance of variables the findings of the present study are more in lines with Rahimi et al. [19]. The results are found to be similar with other international studies like [20–23].

The findings that seem surprising are non-significance of smoking and diabetes. However, similar results concerning diabetes and smoking have been reported in other studies [24–25] as well. The reason behind may be smoking and diabetes are basically causes of heart problem at initial stages. We were only concerned with patients of NYHA class III and IV which are advanced stages of heart failure. Up to these stages, these factors (diabetes and smoking) may probably be controlled by medications and hence these factors do not have significant effect on deaths due to heart failure in class III and IV.

Performance of model was checked using calibration slope and ROC curve. Both concluded in adequacy of model for prediction. ROC curves were also used to discuss the goodness of model with respect to time. Nomogram was used to find the probability of survival by graphical method. It was observed that fall of survival probability was almost same for Kaplan Meier plot and nomogram.

## Conclusion

It can be concluded that growing age, renal dysfunction (having serum creatinine greater than its normal level 1.5), high BP (higher than normal range), higher level of anaemia and lower values of ejection fraction (EF) are the key factors contributing towards increased risk of mortality among heart failure patients. Increased level of serum sodium can reduce the odds of death. No significant differences were found due to smoking status, diabetes and gender of patients.

## Supporting information

S1 Data DATA_MINIMAL. (CSV)
